# The effects of various penalty parameter values in Q.Clear algorithm for rectal cancer detection on ^18^F-FDG images using a BGO-based PET/CT scanner: a phantom and clinical study

**DOI:** 10.1186/s40658-023-00587-y

**Published:** 2023-10-16

**Authors:** Fatemeh Sadeghi, Peyman Sheikhzadeh, Saeed Farzanehfar, Pardis Ghafarian, Yalda Moafpurian, Mohammadreza Ay

**Affiliations:** 1 Department of Medical Physics and Biomedical Engineering, Tehran University of Medical Sciences, Tehran, Iran; 2 Research Center for Molecular and Cellular Imaging (RCMCI), Advanced Medical Technologies and Equipment Institute (AMTEI), Tehran University of Medical Sciences, Tehran, Iran; 3 Department of Nuclear Medicine, Imam Khomeini Hospital Complex, Tehran University of Medical Sciences, Tehran, Iran; 4 Chronic Respiratory Diseases Research Center, National Research Institute of Tuberculosis and Lung Diseases (NRITLD), Shahid Beheshti University of Medical Sciences, Tehran, Iran; 5 PET/CT and Cyclotron Center, Masih Daneshvari Hospital, Shahid Beheshti University of Medical Sciences, Tehran, Iran; 6 Department of Nuclear Medicine, Shiraz University of Medical Sciences, Shiraz, 7134814336 Iran

**Keywords:** Bayesian method, ^18^F-FDG, PET-CT, Image reconstruction

## Abstract

**Background:**

The Q.Clear algorithm is a fully convergent iterative image reconstruction technique. We hypothesize that different PET/CT scanners with distinct crystal properties will require different optimal settings for the Q.Clear algorithm. Many studies have investigated the improvement of the Q.Clear reconstruction algorithm on PET/CT scanner with LYSO crystals and SiPM detectors. We propose an optimum penalization factor (*β*) for the detection of rectal cancer and its metastases using a BGO-based detector PET/CT system which obtained via accurate and comprehensive phantom and clinical studies.

**Methods:**

^18^F-FDG PET-CT scans were acquired from NEMA phantom with lesion-to-background ratio (LBR) of 2:1, 4:1, 8:1, and 15 patients with rectal cancer. Clinical lesions were classified into two size groups. OSEM and Q.Clear (*β* value of 100–500) reconstruction was applied. In Q.Clear, background variability (BV), contrast recovery (CR), signal-to-noise ratio (SNR), SUVmax, and signal-to-background ratio (SBR) were evaluated and compared to OSEM.

**Results:**

OSEM had 11.5–18.6% higher BV than Q.Clear using *β* value of 500. Conversely, RC from OSEM to Q.Clear using *β* value of 500 decreased by 3.3–7.7% for a sphere with a diameter of 10 mm and 2.5–5.1% for a sphere with a diameter of 37 mm. Furthermore, the increment of contrast using a *β* value of 500 was 5.2–8.1% in the smallest spheres compared to OSEM. When the *β* value was increased from 100 to 500, the SNR increased by 49.1% and 30.8% in the smallest and largest spheres at LBR 2:1, respectively. At LBR of 8:1, the relative difference of SNR between *β* value of 100 and 500 was 43.7% and 44.0% in the smallest and largest spheres, respectively. In the clinical study, as *β* increased from 100 to 500, the SUVmax decreased by 47.7% in small and 31.1% in large lesions. OSEM demonstrated the least SUVmax, SBR, and contrast. The decrement of SBR and contrast using OSEM were 13.6% and 12.9% in small and 4.2% and 3.4%, respectively, in large lesions.

**Conclusions:**

Implementing Q.Clear enhances quantitative accuracies through a fully convergent voxel-based image approach, employing a penalization factor. In the BGO-based scanner, the optimal *β* value for small lesions ranges from 200 for LBR 2:1 to 300 for LBR 8:1. For large lesions, the optimal *β* value is between 400 for LBR 2:1 and 500 for LBR 8:1. We recommended *β* value of 300 for small lesions and *β* value of 500 for large lesions in clinical study.

## Introduction

The combination of positron emission tomography (PET) with computed tomography (CT) is commonly employed as a powerful imaging tool in the clinical assessment of oncological issues [1]. There is a growing desire to enhance the spatial resolution in PET scanners to achieve better detectability, particularly for small lesions [2]. The precise determination of radiopharmaceutical's standardized uptake value (SUV) in lesions and body regions is influenced by various factors, among which the reconstruction method plays a pivotal role [3]. Analytical and iterative reconstruction stands as the two primary methods used for image reconstruction [4]. Analytical reconstruction techniques offer a direct mathematical approach to image generation, encompassing filtered back-projection and the Fourier transformation method [5, 6]. Iterative methods involve a more intricate mathematical solution that requires multiple iterations to yield an image. These iterative algorithms enhance image quality by enabling more precise modeling of the data acquisition process [7]. The ordered subset expectation maximization (OSEM) method stands as the most widely utilized iterative reconstruction method in PET imaging [8]. In OSEM, the image noise escalates as the number of iterations increases. To manage the noise, the algorithm is halted before the noise becomes excessive (typically 2–4 iterations) and post-filtered [9, 10]. Due to the limitation on the number of iterations used, each image voxel is incompletely converged; hence, quantitative parameters are not estimated correctly [11, 12].

The block sequential regularized expectation maximization (BSREM) is a Bayesian penalized likelihood (BPL) reconstruction algorithm, marketed under the name Q.Clear (GE Healthcare, Milwaukee, WI, USA [13]. Like other algorithms, Q.Clear incorporates point spread function modeling and, additionally, a relative difference penalty (RDP), which considers the relative difference between neighboring voxels to prevent excessive smoothing along significant edges [14, 15]. The influence of RDP is regulated by a user-defined parameter referred to as the penalization factor (*β* value), which governs the overall degree of noise suppression in Q.Clear, thereby allowing for numerous iterations without a corresponding increase in noise [16]. A higher number of iterations facilitates complete convergence for each individual voxel, enabling accurate estimation of quantitative parameters. It is worth noting that an improper optimization of the beta factor leads to poor image quality and diminished lesion detection [17].

Lutetium oxyorthosilicate (LSO) and lutetium yttrium oxyorthosilicate (LYSO) detectors are currently favored in commercial time of flight (TOF) systems due to their quicker decay times and shorter coincidence timing windows. Bismuth germanium oxide (BGO) detectors possess a broader coincidence window, rendering the incorporation of TOF technology into a PET system utilizing BGO crystals unfeasible. The BGO timing resolution is lower than that for LYSO and LSO, due to its longer decay time and lower light output. This deficiency contributes to higher scatter and elevated noise levels within the BGO scanner. Despite this, BGO crystals still offer specific advantages, such as a higher effective atomic number that results in increased detection efficiency, lower intrinsic radiation, and reduced production costs. However, their utilization necessitates reconstruction enhancement procedures [18]. A PET system's sensitivity is pivotal in achieving adequate counting statistics for a favorable signal-to-noise ratio during image reconstruction [19]. Our study's hypothesis is that various scanners, distinguished by different attributes most notably crystal type, coincidence timing window, and FOV will yield disparate images. Consequently, a comprehensive exploration of each scanner becomes imperative to evaluate the image quality and quantification performance of the Q.Clear reconstruction algorithm. A plethora of prior research has investigated the efficacy of Q.Clear in enhancing ^18^F-FDG PET imaging on scanners using LYSO crystals and integrating TOF technology [20–23].

For instance, Lindström et al. analyzed data from a NEMA image quality phantom and clinical whole-body scans, including 68Ga-DOTATOC (*n* = 13), 18F-fluoride (*n* = 10), and 11C-acetate (*n* = 13). The data were acquired using a GE Discovery MI equipped with LYSO crystals, and reconstructed images were generated through both OSEM and Q.Clear methods, using *β* values of 133, 267, 400, and 533 (both algorithms incorporating TOF and PSF techniques). In tracer-specific ranges of *β* values, Q.Clear reconstruction yielded higher SNR (a minimum of 25%) and SBR (a maximum of 23%) compared to conventional OSEM reconstruction. Comparable levels of SNR, SBR, and noise could be achieved with BSREM in shorter acquisition times or with lower administered dosages, contrary to what OSEM could achieve[22].

Ahn et al. examined 25 patients and NEMA, anthropomorphic, and oval phantoms scanned with LYSO crystals on a GE Discovery PET/CT 690 scanner. Images were reconstructed with Q.Clear with a *β* value of 350 and OSEM. Their findings indicated enhanced accuracy in lesion quantitation for Q.Clear in comparison with OSEM, with a notable improvement observed in regions with low uptake, such as the lungs [21]. Our study was conducted using a non-time of flight discovery IQ PET/CT scanner equipped with BGO crystals and a 26-cm axial field of view (FOV).

To the best of our knowledge, Q.Clear's performance using different *β* values in terms of lesion size at various activity levels has not been extensively undertaken. Our previous research exclusively conducted a phantom study at only a lesion-to-background ratio of 4 (LBR 4:1) to evaluate Q.Clear in 68Ga-PSMA PET-CT examinations. We concluded that at LBR 4:1, Q.Clear with a *β* value of 400 is most effective for reconstructing small lesions, while larger lesions in the phantom benefit from *β* values of 600 [24]. Chen et al. analyzed data from a NEMA/IEC image quality phantom with a concentration ratio of 4:1 for ^18^F-FDG solution in spheres. The data were acquired using a 3-ring DMI scanner and were reconstructed using Q.Clear with *β* values ranging from 100 to 500 at 100-point intervals, in contrast to OSEM. The target-to-background ratio (TBR) and contrast noise ratio (CNR) exhibited an increase corresponding to the higher beta value, whereas the noise demonstrated an inverse trend, except for the smallest sphere (10 mm diameter). For the smallest hot sphere, the TBR reached a plateau when the beta value reached 300, while the CNR gradually declined thereafter [25]. This study aimed to thoroughly investigate Q.Clear reconstruction by conducting an extensive analysis utilizing a combination of phantom and clinical studies. In the context of ^18^F-FDG radiopharmaceutical studies, the lesion-to-background ratio emerges as a crucial parameter for consideration. We incorporated three different lesion-to-background ratios in our phantom study, facilitating the evaluation of diverse lesion sizes while simulating various focal point uptakes.

Discovery IQ PET/CT scanners, which utilize BGO crystals, have gained worldwide recognition and acceptance among clinicians. The next generation of Discovery IQ has been meticulously engineered and designed to function as a scalable, high-performance diagnostic system capable of delivering exceptional image quality while employing a reduced dose. However, due to the widespread adoption of BGO crystals, specialized studies pertaining to these crystals are imperative. Our study evaluated the influence of distinct penalty functions within the Q.Clear algorithm on ^18^F-FDG PET-CT images using a non-time-of-flight PET/CT system based on BGO crystals. The quantitative evaluation encompassed different penalization factors (*β*) for both phantom and clinical data across various LBRs.

## Methods

### Phantom study

The images were acquired using our department's GE Discovery IQ PET-CT system (GE Healthcare, USA). The scanner features five detector rings with 36 detector units per ring. Each detector is comprised of 6.3 × 6.3 × 30 mm BGO crystals. The scanner has a 70-cm trans-axial field of view (FOV) and a 26-cm axial FOV. It includes a 16-slice CT scanner. The PET images were reconstructed using Q.Clear and OSEM + PSF. According to previous studies, for oncology whole-body ^18^F-FDG scans, the *β* value is typically set within the range of 400–450 [20, 23, 26, 27]. The optimal number of iterations for full convergence, as suggested by the manufacturer, is 25. Additionally, the manufacturer recommends the OSEM algorithm with 4 iterations, 24 subsets, and 4.8 mm Gaussian post-filtering for standard clinical image reconstruction using ^18^F-FDG. Thus, in our study, Q.Clear reconstruction employed 25 iterations and *β* values ranging from 100 to 500 in intervals of 100. OSEM + PSF (routine protocol), referred to as OSEM in this paper, was applied according to the manufacturer's suggestion [28].

A National Electrical Manufacturers Association (NEMA) image quality phantom with a total volume of 9780 ml and a height of 19.4 cm was utilized [29]. This phantom contains six spheres with diameters of 10, 13, 17, 22, 28, and 37 mm [30]. The spheres were filled with an ^18^F-FDG solution to achieve lesion-to-background ratios (LBR) of 2:1, 4:1, and 8:1. The background was filled with activity concentrations of 5.1, 4.9, and 5 KBq/ml of ^18^F-FDG, respectively. A lung insert at the center of the phantom with an LBR of 8:1 was incorporated to simulate human lung tissue. This insert was filled with water and Styrofoam.

**Fig. 1:**
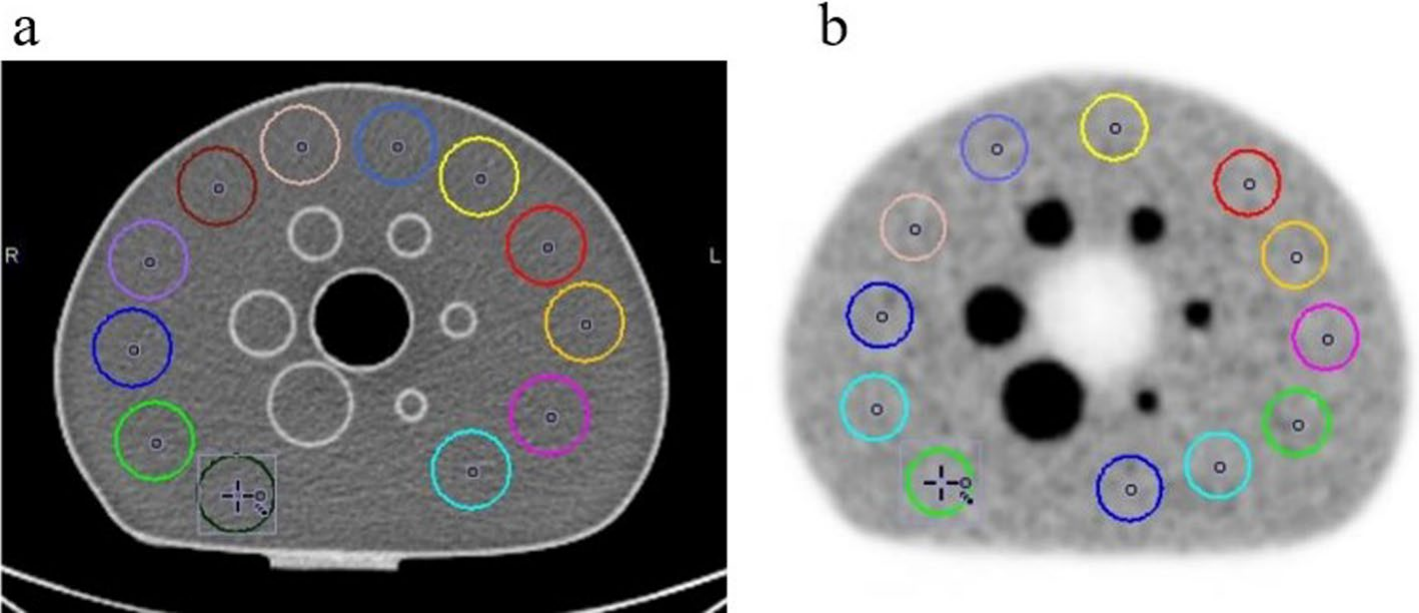
Central transfer slice of the NEMA image quality phantom acquired by **a** CT scanner and **b** PET scanner. The 12 regions of interest (ROIs) with a 5 mm diameter drawn on the background are shown for each image

According to NEMA phantom analysis standards, metrics such as maximum standard uptake value (${\mathrm{SUV}}_{\mathrm{max}}$), contrast recovery (CR), recovery coefficient (RC), background variability (BV), lung residual error (LE), contrast, and signal-to-noise ratio (SNR) were measured. Each image was analyzed using PMOD software (version 3.8, developed by PMOD-Technologies LLC, Zurich, Switzerland) [16, 31, 32]. For image assessment in the PMOD, the slice traversing the central region of the spheres is identified. In this slice and two slices before and after, 12 ROIs with a diameter of 10 mm are delineated in each slice within the background area. Therefore, a total of 60 ROIs are considered in the phantom's background region. The ROIs are drawn, ensuring their distance from both the spheres and the phantom edge is no less than 15 mm (Fig. 1). To incorporate spherical regions, the volume of interest (VOI) was positioned within the central slice of spheres, with each sphere having a diameter proportionate to its individual sphere diameter.

CR was defined as the following:

1 $${\text{CR}} = \frac{{\frac{{C_{{\text{H}}} }}{{C_{{\text{B}}} }} - 1}}{{\frac{{a_{{\text{H}}} }}{{a_{{\text{B}}} }} - 1}},$$

where ${C}_{\mathrm{H}}$ is the mean count in hot spheres,${C}_{\mathrm{B}}$ is the mean count in the background, and $\frac{{a}_{\mathrm{H}}}{{a}_{\mathrm{B}}}$ represents the target ratio of the hot activity. RC is defined as the ratio of measured activity to the true activity of each insert. The coefficient of variation (COV) is used as a metric for describing BV in the image [33]. BV is calculated as the ratio of SD to the mean activity in 60 background regions of interest (ROIs) [26]. The LE is determined as the ratio of mean activity in lung insert to mean activity in background ROIs.

Contrast is determined as the mean voxel value in each sphere divided by the mean voxel value of the background (BG) ROIs, and SNR is the ratio of the mean voxel count of each sphere to the SD of BG ROIs.

To compare the effects of different *β* values, the relative changes in each assessment parameter were examined at consistent intervals of *β* values. For this purpose, the relative difference between reconstruction a and reconstruction b was calculated as:

2 $$\Delta {\text{feature}}_{{\left( {a - b} \right)}} \% = \frac{{{\text{feature}}_{a} - {\text{feature}}_{b} }}{{{\text{feature}}_{a} }} *100$$

### Clinical study

We assessed the three-dimensional whole-body ^18^F-FDG PET-CT scans of 15 patients (11 men and 4 women) diagnosed with rectal cancer. The patients, with a weight range of 45–100 kg, a median height of 160 cm, and a median age of 60 years, were intravenously administrated an activity concentration of 3.5–5 MBq of ^18^F-FDG per kilogram of body weight. All patients fasted for six hours before the injection, and the uptake time range was 60 ± 3.0 min. Images were captured with an acquisition time of 3 min per bed position (min/bp).

For the purpose of clinical image assessment, all lesions were identified by two nuclear medicine specialists and were subsequently enclosed with a volume of interest (VOI) to encompass them entirely. In the clinical data, we examined primary tumor in rectum and rectal cancer metastases spread throughout the lung and pelvic (15 primary rectal tumors, 12 lung metastases, and 18 pelvic metastatic lesions). These 45 lesions were assessed and categorized into two size groups: 27 small lesions (diameter ⩽ 10 mm) and 18 large lesions (diameter > 10 mm). Table 1 illustrates the mean activity of individual lesions alongside their respective tumor types, categorized into two size groups. For the quantitative analysis, nine ROIs with a diameter of 30 mm were positioned in the three largest slices of the right lobe of the liver [34]. The liver noise was calculated as the ratio of the standard deviation to the mean activity of ROIs in the liver. SNR was defined as the lesion ${\mathrm{SUV}}_{\mathrm{max}}$ divided by liver noise, and signal-to-background ratio (SBR) was calculated as the lesion ${\mathrm{SUV}}_{\mathrm{max}}$ divided by ${\mathrm{SUV}}_{\mathrm{mean}}$ of the liver.

**Table 1:** Mean activity of 45 lesions in a clinical study across six reconstruction methods (Q.Clear with *β* values of 100, 200, 300, 400, 500, and OSEM), categorized by lesion size: 27 small lesions (diameter ⩽ 10 mm) and 18 large lesions (diameter > 10 mm), and individual tumor type identification (primary rectal tumor, lung metastasis, pelvic metastasis)

Small lesions (diameter ⩽ 10 mm)							
Lesion	*β* value = 100	*β* value = 200	*β* value = 300	*β* value = 400	*β* value = 500	OSEM	Tumor type
1	14.62	14.05	13.5	13.05	12.63	10.94	Primary rectal tumor
2	33.66	32.93	32.29	31.7	31.16	28.24	Primary rectal tumor
3	26.59	25.73	24.97	24.24	23.55	19.91	Primary rectal tumor
4	33.85	32.94	32.16	31.41	30.71	26.34	Primary rectal tumor
5	33.2	30.52	28.26	26.26	24.49	19.87	Primary rectal tumor
6	19.71	19.17	18.65	18.16	17.7	15.57	Primary rectal tumor
7	16.6	16	15.4	14.82	14.29	14.26	Primary rectal tumor
8	36.4	35.88	35.36	34.83	34.31	30.98	Lung metastasis
9	26.29	25.62	24.99	24.37	23.78	22.29	Lung metastasis
10	11.35	10.96	11.71	10.57	10.21	9.11	Lung metastasis
11	12.05	11.59	12.48	11.14	10.74	9.72	Lung metastasis
12	13.61	12.59	14.88	11.75	11.04	9.18	Lung metastasis
13	18.59	16.93	20.68	15.62	14.58	12.85	Lung metastasis
14	17.56	17.35	17.12	16.86	16.59	15.5	Lung metastasis
15	17.37	16.74	16.21	15.74	15.34	14.01	Lung metastasis
16	12.27	12.01	11.74	11.49	11.22	10.29	Pelvic metastasis
17	14.054	13.82	13.59	13.37	13.14	12.29	Pelvic metastasis
18	19.86	17.69	15.8	14.27	13.017	10.04	Pelvic metastasis
19	18.4	17.71	16.32	15.78	15.25	13.16	Pelvic metastasis
20	13.92	13.62	12.65	12.24	11.88	11.02	Pelvic metastasis
21	21.52	18.42	17.74	17.39	17.01	16.33	Pelvic metastasis
22	23.08	21.07	19.75	18.78	18.43	17.55	Pelvic metastasis
23	25.32	22.34	21.27	20.73	20.27	18.67	Pelvic metastasis
24	19.39	16.78	15.43	14.25	13.2	11.42	Pelvic metastasis
25	14.92	14.65	14.25	13.79	13.46	12.31	Pelvic metastasis
26	17.05	14.1	12.59	11.68	10.87	11.22	Pelvic metastasis
27	40.26	38.34	36.77	35.48	34.22	27.96	Pelvic metastasis

Large lesions (diameter > 10 mm)							
Lesion	*β* value = 100	*β* value = 200	*β* value = 300	*β* value = 400	*β* value = 500	OSEM	Tumor type
1	30.71	30.27	29.84	29.45	29.07	29.59	Primary rectal tumor
2	21.92	21.4	21.02	20.72	20.46	20.78	Primary rectal tumor
3	34.89	34.08	33.35	32.67	32.04	31.56	Primary rectal tumor
4	30.22	29.79	29.42	29.1	28.81	27.5	Primary rectal tumor
5	27.51	27.29	27.06	26.86	26.66	25.77	Primary rectal tumor
6	8.99	8.87	8.78	8.67	8.51	7.34	Primary rectal tumor
7	9.06	8.94	8.84	8.74	8.62	8.28	Primary rectal tumor
8	8.22	7.91	7.28	6.98	6.88	6.23	Primary rectal tumor
9	17.36	17.83	17.3	17.27	17.24	16.62	Lung metastasis
10	18.05	18.27	17.8	17.55	17.31	16.51	Lung metastasis
11	43.14	42.06	41.41	40.99	40.7	38.29	Lung metastasis
12	22.7	20.23	19.35	18.65	18.06	19.85	Lung metastasis
13	15.79	14.29	13.35	12.65	12.12	14.16	Pelvic metastasis
14	32.43	28.86	26.9	26.17	25.44	26.89	Pelvic metastasis
15	26.21	23.14	21.69	20.67	19.87	19.5	Pelvic metastasis
16	36.17	35.03	34.08	33.26	32.44	30.64	Pelvic metastasis
17	10.06	8.7	8.042	7.6	7.26	6.31	Pelvic metastasis
18	16.39	12.39	10.53	9.58	8.92	8.55	Pelvic metastasis

### Statistical analysis

We employed paired Student *t* test in both our phantom and clinical studies. We conducted the *t* test individually for each evaluation parameter. This involved comparisons between two distinct reconstruction approaches, either comparing results derived from different *β* values or contrasting outcomes from a single *β* value with those from OSEM reconstruction. Statistical variances in image evaluation were assessed using the analysis of variances (ANOVA), followed by post hoc Bonferroni corrections to adjust comparisons among multiple reconstructions. *P* value < 0.05 was set as the significance level [35]. Statistical analyses were conducted using the SPSS software version 23.0 (IBM Corporation, Armonk, NY, USA).

## Results

### NEMA phantom

**Fig. 2:**
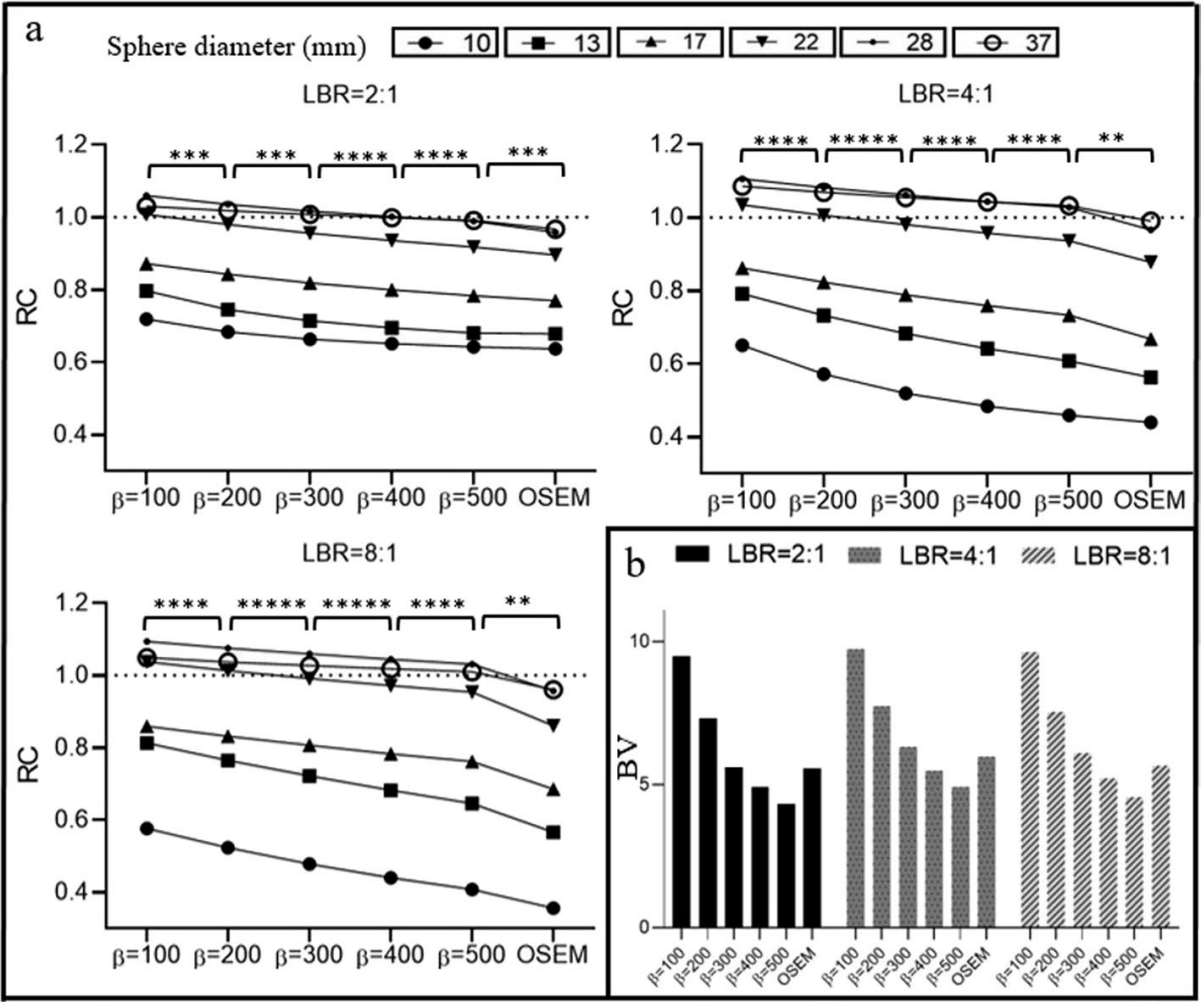
**a** A schematic comparison of hot spheres of different sizes (10 mm, 13 mm, 17 mm, 22 mm, 28 mm, and 37 mm) in RC versus different reconstruction algorithms (with OSEM and Q.Clear employing *β* values ranging from 100 to 500 at 100-point intervals). The data were acquired using a NEMA image quality phantom filled with lesion-to-background ratios (LBR) of 2:1, 4:1, and 8:1. **b** Results for background variability in OSEM and Q.Clear using the same *β* values (100–500) and LBRs. Note that the dashed line depicted the optimal RC, which is equal to 1. The asterisks in the figures symbolize the p values resulting from the paired *t* test conducted between the two reconstruction methods, wherein * indicates *p* < 0.01, ** indicates *p* < 0.001, *** indicates *p* < 0.0001, **** indicates *p* < 0.00001 and ***** indicates *p* < 0.000001

**Fig. 3:**
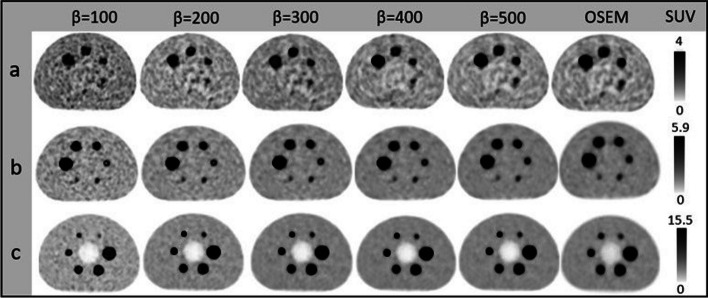
The central transverse slice of NEMA IEC phantom, from left to right, reconstructed by Q.Clear with *β* values ranging from 100 to 500 at 100-point intervals and OSEM and from top to bottom were filled with **a** Background activity concentrations of 5.1 KBq/ml and LBR = 2:1, **b** Background activity concentrations of 4.9 KBq/ml and LBR = 4:1, **c** Background activity concentrations of 5 KBq/ml and LBR = 8:1

Figure 2 illustrates the results of BV and RC in phantom studies with LBRs of 2:1, 4:1, and 8:1. With Q.Clear reconstructions, BV increased as the *β* value decreased. The $\Delta$ ${\mathrm{BV}}_{(200-100)}\%$ were − 39.7%, − 35.6%, and − 38.5%, and $\Delta$ ${\mathrm{BV}}_{(500-400)}\%$ were − 11.2%, − 13.3%, and − 14.7% for LBRs of 2:1, 4:1, and 8:1, respectively. OSEM presented 11.5%, 18.6%, and 17.0% higher BV than ${\mathrm{Q}.\mathrm{Clear }}_{500}$. A decreasing *β* value escalated the RC. The percentage changes between OSEM and ${\mathrm{Q}.\mathrm{Clear}}_{500}$ were − 3.3%, − 5.1%, and − 7.7% for the smallest sphere (10 mm) and − 2.5%, − 4.2%, and − 5.1% for the largest sphere (37 mm) at LBRs of 2:1, 4:1, and 8:1. Figure 3 displays NEMA phantom slices reconstructed with Q.Clear and OSEM for varying LBRs and *β* values. Elevated noise in ${\mathrm{Q}.\mathrm{Clear }}_{100}$ compromised the image quality. Higher *β* values improved homogeneity for low LBRs, whereas lower *β* values were preferable for high LBRs compared to their low counterparts.

**Fig. 4:**
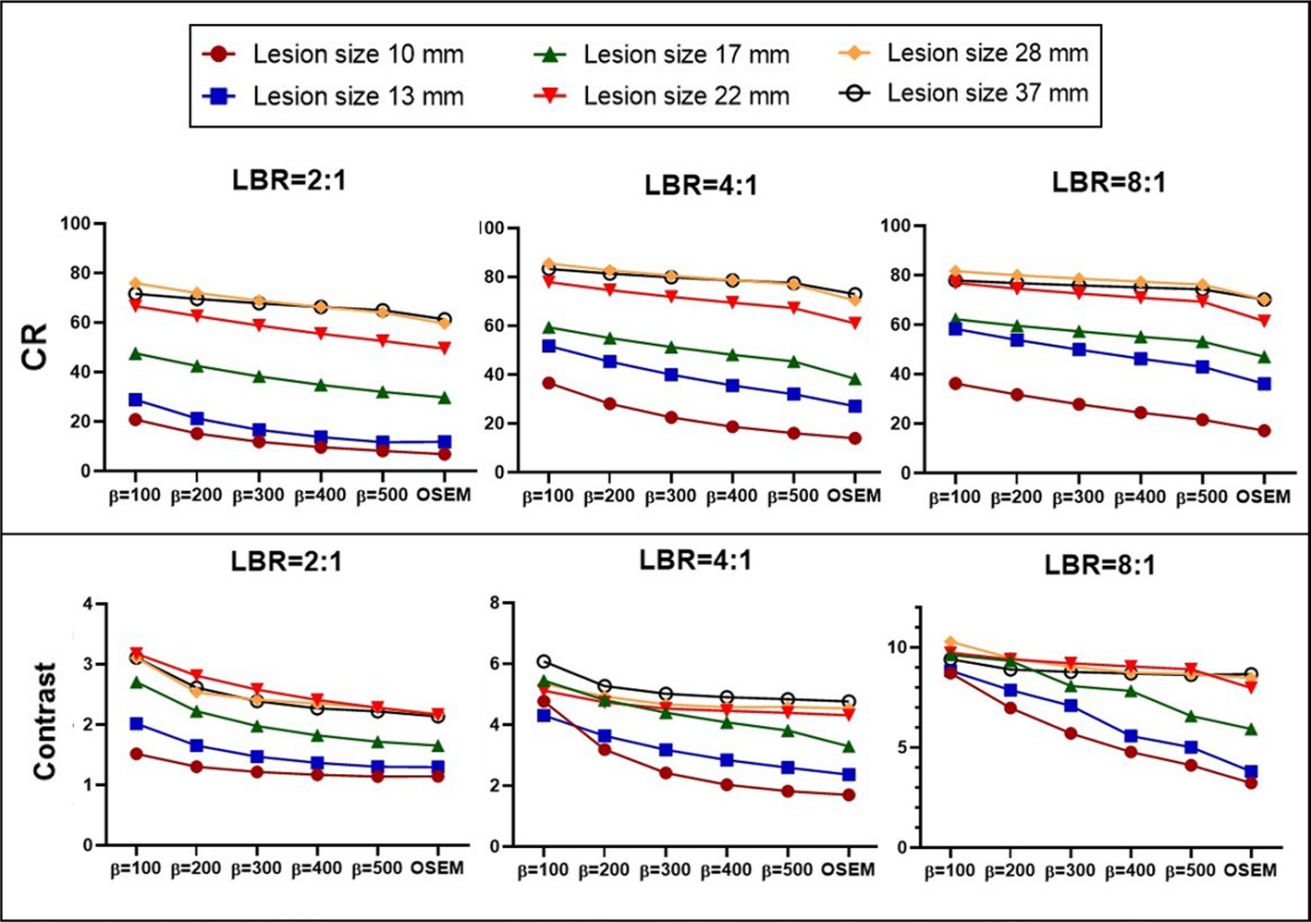
CR and contrast in hot spheres with diameters of 10 mm, 13 mm, 17 mm, 22 mm, 28 mm, and 37 mm at LBRs of 2:1, 4:1, and 8:1. These were reconstructed using OSEM and Q.Clear with *β* values of 100, 200, 300, 400, and 500. The *p* values for paired *t* tests between reconstruction methods (including six different lesion sizes) were *p* < 0.05

Figure 4 showcases the CR and contrast for various sphere sizes and LBRs in phantom studies. There was a negative correlation between *β* values and lesion CR. Q.Clear consistently demonstrated higher CR and contrast compared to OSEM across all sphere dimensions and LBRs. For instance, at LBR 2:1, OSEM's CR values were 19.1%, 10.3%, and 6.1% lower than ${\mathrm{Q}.\mathrm{Clear}}_{500}$ for 10 mm, 22 mm, and 37 mm spheres, respectively. The relative contrast difference expanded with diminishing *β* values. At LBRs of 2:1, 4:1, and 8:1, OSEM had percentage disparities of − 8.1%, − 7.0%, and − 5.2%, compared to ${\mathrm{Q}.\mathrm{Clear}}_{500}$ for the 10 mm sphere. Refer to Fig. 4 for more detailed results.

There was an inverse correlation between LE and *β* value. At LBR of 8:1, the LE values stood at 14.6 for ${\mathrm{Q}.\mathrm{Clear}}_{100}$ and 28.4 for OSEM. The lowest SNR was observed for ${\mathrm{Q}.\mathrm{Clear}}_{100}$. The percentage SNR difference between ${\mathrm{Q}.\mathrm{Clear}}_{500}$ and ${\mathrm{Q}.\mathrm{Clear}}_{100}$ was 49.1% for the smallest sphere (10 mm) and 30.8% for the largest sphere (37 mm) at an LBR of 2:1. Increasing the LBR did yield a meaningful difference (*p* > 0.05) in SNR across different lesion sizes. At an LBR of 8:1 (according to Table 2), the percentage SNR difference between the ${\mathrm{Q}.\mathrm{Clear}}_{500}$ and ${\mathrm{Q}.\mathrm{Clear}}_{100}$ was 43.7% for the smallest sphere (10 mm) and 44.0% for the largest sphere (37 mm). For spheres sized 10 mm, 22 mm, and 37 mm, OSEM had 30.6%, 20.4%, and 18.8% reduced SNR than ${\mathrm{Q}.\mathrm{Clear}}_{500}$.

**Table 2:** The relative difference in SNR among distinct *β* values in Q.Clear reconstruction for hot lesions measuring 10 mm, 22 mm, and 37 mm in diameter, filled with LBRs of 2:1, 4:1, and 8:1 (Above)

LBR	Lesion size	Relative difference of SNR(%)					
		$\Delta {\mathrm{SNR}}_{\left(200-100\right)}$	$\Delta {\mathrm{SNR}}_{(300-200)}$	$\Delta {\mathrm{SNR}}_{\left(400-300\right)}$	$\Delta {\mathrm{SNR}}_{\left(500-400\right)}$	$\Delta {\mathrm{SNR}}_{\left(500-100\right)}$	$\Delta {\mathrm{SNR}}_{\left(\mathrm{OSEM}-500\right)}$
2:1	10 mm	26.5	16	11.4	8.8	49.1	− 4.9
	22 mm	24.9	12.8	6.9	3.9	42.8	− 0.9
	37 mm	18.3	8.4	4.7	2.9	30.8	1.2
	p	S	S	S	S	S	S
4:1	10 mm	26.1	14.8	9.5	7.3	45.1	− 10.2
	22 mm	24.6	12.2	6.8	6.9	40.5	− 7.3
	37 mm	21.7	10.6	6.4	4.7	37.4	− 3.1
	p	S	S	S	NS	S	S
8:1	10 mm	25.2	13.6	8.7	6	43.7	− 30.6
	22 mm	24.2	13.5	8.3	5.6	40.7	− 20.4
	37 mm	22.1	12.5	8	5.4	44.0	− 18.8
	p	S	S	S	NS	S	S
*Relative difference of LE(%)*							
8:1	Lung insert	$\Delta {\mathrm{LE}}_{\left(200-100\right)}$	$\Delta {\mathrm{LE}}_{\left(300-200\right)}$	$\Delta {\mathrm{LE}}_{(400-300)}$	$\Delta {\mathrm{LE}}_{\left(500-400\right)}$	$\Delta {\mathrm{LE}}_{\left(500-100\right)}$	$\Delta {\mathrm{LE}}_{\left(\mathrm{OSEM}-500\right)}$
		− 5.3	− 4.1	− 3.8	− 3.2	− 13.1	54.5
	p	S	S	NS	NS	S	S

Additionally, the relative difference in lung residual error between OSEM and Q.Clear in the lung insert of the NEMA IEC phantom at the LBR of 8:1 is presented (Below). *P* values were deduced using a paired *t* test. ‘*S*’ denotes statistically significant differences (*p* value ≤ 0.05), whereas 'NS' indicates no significant variation (*p* value > 0.05).

### Clinical study

**Fig. 5:**
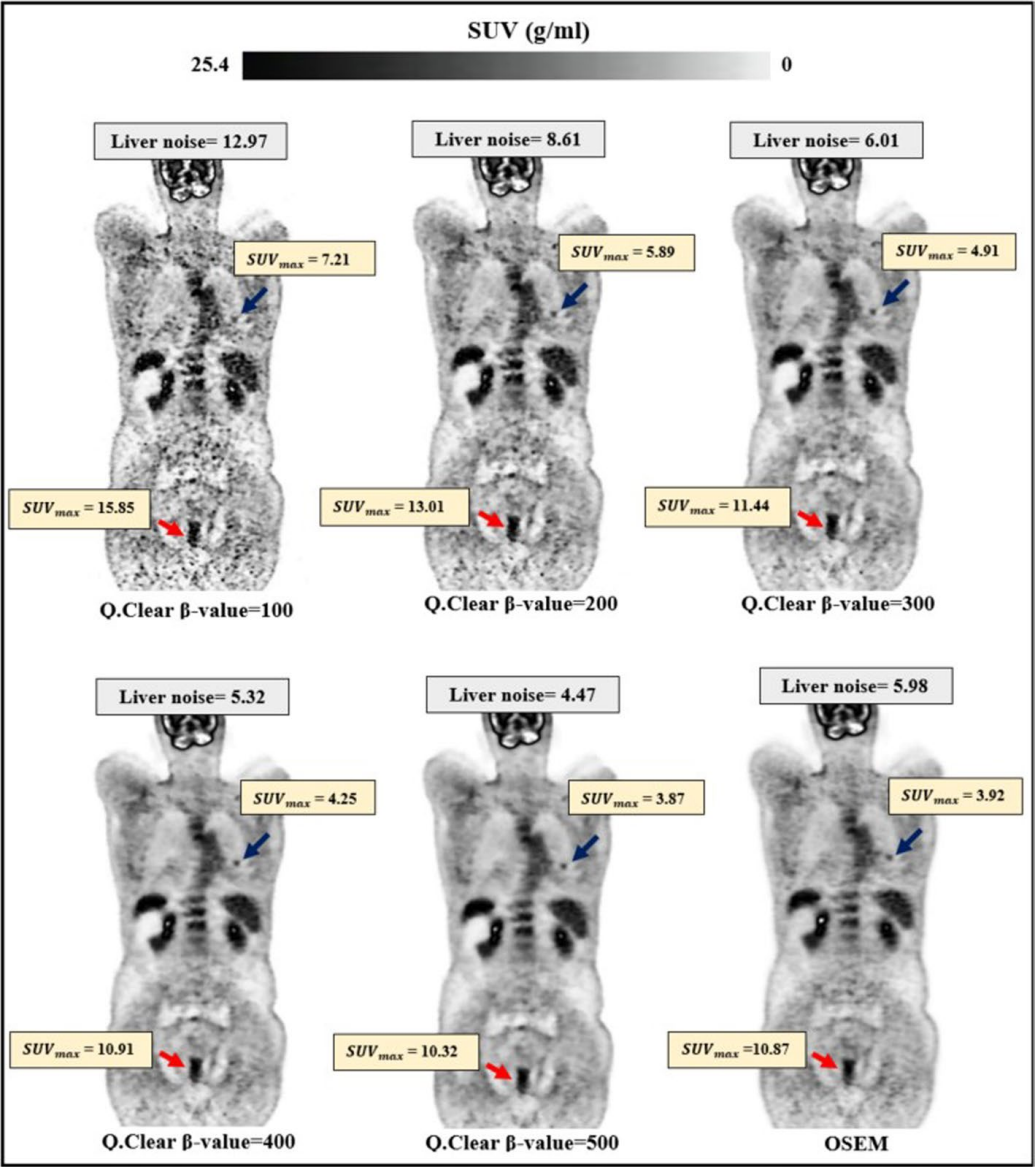
Cross-sectional view of a 58-year-old rectal cancer patient (68 kg, 155 cm), receiving 4 MBq/kg of ^18^F-FDG intravenously. The OSEM reconstruction was utilized with four iterations and 24 subsets with 4.8 mm Gaussian post-filtering. Twenty-five iterations with *β* value of 100, 200, 300, 400, and 500 were used in Q.Clear reconstruction. In each image, the blue arrow points to a lung metastasis with diameter ⩽ 10 mm (small lesion), while the red arrow indicates a primary rectal tumor with diameter > 10 mm (large lesion). The SUVmax of the lesions and the noise in the liver are displayed for each reconstruction

**Fig. 6:**
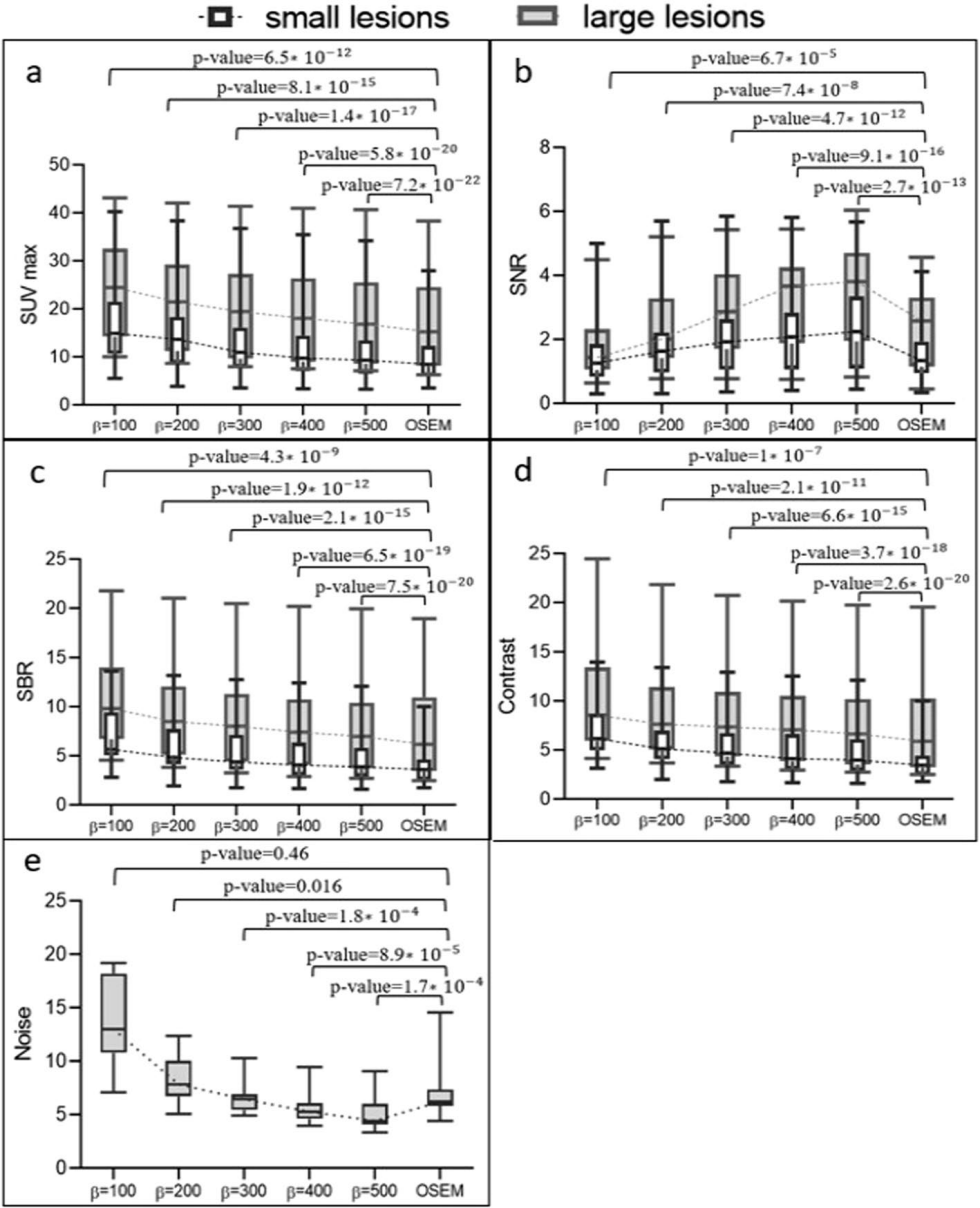
**a** The average SUVmax, **b** SNR, **c** SBR, **d** image contrast, and **e** noise within the uniform liver area for two categories of lesion sizes (small and large) in a medical research using ^18^F-FDG. The reconstruction methods used were OSEM and Q.Clear, with *β* values ranging from 100 to 500 in increments of 100. Dotted lines link the median values. Note that the values depicted in the graphs correspond to the p values derived from the paired *t* test conducted between the two reconstruction techniques

Figure 5 showcases the coronal view of a patient with rectal cancer. It reveals that lower *β* values degrade image quality and the signal-to-noise ratio (SNR) due to amplified noise. Conversely, elevated *β* values enhance image clarity and SNR by proficiently diminishing noise. Figure 6 presents a quantitative analysis of 45 lesions across all reconstruction algorithms. It demonstrates that escalating *β* values precipitate a reduction in both ${\mathrm{SUV}}_{\mathrm{max}}$ and noise. The percentage difference in ${\mathrm{SUV}}_{\mathrm{max}}$ between ${\mathrm{Q}.\mathrm{Clear}}_{500}$ and ${\mathrm{Q}.\mathrm{Clear}}_{100}$ stood at − 47.7% for smaller lesions and − 33.1% for larger lesions. OSEM exhibited a reduced ${\mathrm{SUV}}_{\mathrm{max}}$ compared to all other reconstruction methods. Consequently, the percentage difference in ${\mathrm{SUV}}_{\mathrm{max}}$ between OSEM and ${\mathrm{Q}.\mathrm{Clear}}_{500}$ reconstructions decreased by 12.0% for smaller lesions and 3.6% for larger lesions. The mean noise levels registered at 13.3 ± 3.9 for ${\mathrm{BSREM}}_{100}$ and 5 ± 1.5 for ${\mathrm{BSREM}}_{500}$. The relative noise difference between OSEM and ${\mathrm{Q}.\mathrm{Clear}}_{500}$ amounted to 35.0%.

There was a positive correlation between *β* values and SNR, contrasting the trends observed in SBR and contrast. For small lesions, *β* values exceeding 100, and for larger lesions, *β* values surpassing 200 yielded a higher SNR compared to OSEM. The percentage difference in SNR between OSEM and ${\mathrm{Q}.\mathrm{Clear}}_{500}$ reconstructions decreased by 57.7% for small lesions and 38.9% for large lesions. Both small and large lesion groups displayed the lowest SBR and contrast values with OSEM, while ${\mathrm{Q}.\mathrm{Clear}}_{100}$ showed the highest. The mean SBRs for ${\mathrm{Q}.\mathrm{Clear}}_{100}$ and ${\mathrm{Q}.\mathrm{Clear}}_{500}$ were 6.9 ± 2.7 and 4.6 ± 2.3 for small lesions, and 10.4 ± 5 and 7.6 ± 4.6 for large lesions, respectively. The contrast of small and large lesions on ${\mathrm{Q}.\mathrm{Clear}}_{500}$ diminished by 52.6% and 40.3% compared to ${\mathrm{Q}.\mathrm{Clear}}_{100}$. A statistically significant variance (*p* < 0.001) was observed in measured SBR and contrast between OSEM and Q.Clear. The percentage deviations in SBR and contrast between OSEM and ${\mathrm{Q}.\mathrm{Clear}}_{500}$ reconstructions were − 13.6% and − 12.9% for lesions ⩽10 mm in diameter, and − 4.2% and − 3.4% for lesions > 10 mm in diameter, respectively.

## Discussion

This investigation provided both quantitative and qualitative assessments of the Q.Clear reconstruction algorithm, contrasting a range of penalization factors (*β* value) with the prevalent OSEM + PSF algorithm (dubbed OSEM). Analyses were executed on NEMA phantoms filled with varying LBRs and on patients with rectal cancer administered with an ^18^F-FDG radiopharmaceutical.

The adeptness of Q.Clear to curb potential noise surges facilitates the pragmatic use of heightened iterations (about 25 in Q.Clear against 4 in OSEM). Q.Clear reconstructions exhibited superior ${\mathrm{SUV}}_{\mathrm{max}}$, stemming from an increased iteration count and enhanced convergence. High noise in lower *β* values compromises image quality despite their precise quantification. Conversely, enhanced lesion visibility and consistent cold background were achieved with larger *β* values (Fig. 3). The BV for a *β* value of 300 is 5.8 ± 0.4, closely aligned with the BV of OSEM of 5.4 ± 0.4. The outcomes with NEMA phantom are similar to previous findings using NEMA, oval, and anthropomorphic phantom where a *β* value of 350 parallels OSEM in noise levels [3]. However, this contrasts subtly with Bjöersdorff et al.'s findings, who noted that OSEM reconstruction exhibited noise levels akin to a *β* of 550 at 1.5-min frames and *β* at 2.0-min frames [36].

Comparing OSEM and Q.Clear at matched noise levels (*β* = 300), Q.Clear-constructed images exhibited quantitative superiority over OSEM. For instance, measurements in a 22 mm sphere at an LBR of 4:1 indicated decreases of 21.7% in Δ${\mathrm{CR}}_{(\mathrm{OSEM}-300)}\%$, Δ ${\mathrm{contrast}}_{\left(\mathrm{OSEM}-300\right),}$ and 5.2% in Δ${{\mathrm{SUV}}_{\mathrm{max}}}_{(\mathrm{OSEM}-300)}$. Similar trends appeared in our clinical datasets. Caribé et al. substantiated that Q.Clear outperforms OSEM in tumor${\mathrm{SUV}}_{\mathrm{mean}}$, ${\mathrm{SUV}}_{\mathrm{max}}$, and contrast, even when the noise levels are equivalent [17].

The variation in BV resulting from reconstruction with different *β* values did not exhibit significant differences across various LBRs. The relative difference of BV demonstrated a difference of < 4.1% between LBRs of 2:1 and 8:1 and 3.9% between LBRs of 4:1 and 8:1. This was associated with an insignificant variation between background concentrations in the three LBR examinations (Fig. 2). Reynes-Llompart et al.'s [37] study, which utilized a NEMA phantom with LBRs 2:1, 4:1, and 8:1, reported a 1–2% variance in BV among three LBRs. However, we identified distinct trends for contrast, CR, and ${\mathrm{SUV}}_{\mathrm{max}}$, where this relative difference more pronounced in lower LBRs (*p* < 0.05). A prior study indicated that, in the case of cold lesions in the phantom, the CNR also rises with an increase in the *β* value, even if the LBR does not seem to influence it [37].

In Q.Clear, relative differences in ${\mathrm{SUV}}_{\mathrm{max}}$, CR and contrast were more significant for smaller lesions, attributed to the incorporation of PSF modeling into this algorithm. $\Delta {\mathrm{CR}}_{(500-100)}$% dropped by 155.2, 128.8, and 68.1 for the smallest sphere (10 mm); by 26.8, 15.8, and 10.9 for the mid-sized (22 mm); and by 10.3, 7.5, and 4.8 for the largest sphere (37 mm) when LBRs of 2:1, 4:1, and 8:1 were considered, respectively. Our clinical study mirrored these observations. The escalation in quantitative parameters in ${Q.Clear}_{500}$ for small lesions was at least double that of large lesions (Fig. 6). For instance, the relative difference in SNR, SBR, contrast, and ${\mathrm{SUV}}_{\mathrm{max}}$ between OSEM and ${\mathrm{Q}.\mathrm{Clear}}_{500}$ decreased by 57.7%, 13.6%, 12.9%, and 12.0% for small lesions, and by 38.9%, 4.2%, 3.4%, and 3.6% for large lesions.

In our phantom study with an LBR of 8:1, all Q.Clear reconstructions yielded significantly lower LE than OSEM. Thus, $\Delta {\mathrm{LE}}_{\mathrm{OSEM}-500}\mathrm{\%}$ increased by 54.5 (*p* < 0.05, Table 2). Our findings align with those of Elin Lindström. They found that LE was elevated for OSEM compared to *β* values of 133, 267, 400, and 533 [26].

Both phantom and clinical data indicated that quantitative parameters shifted rapidly as the *β* value escalated to 300. Nevertheless, minor alterations at high *β* values were reported. $\Delta {{\mathrm{SUV}}_{\mathrm{max}}}_{200-100}\mathrm{\%}$, $\Delta {\mathrm{contrast}}_{(200-100)}\mathrm{\%}$ and $\Delta {\mathrm{CR}}_{(200-100)}\mathrm{\%}$ dwindled by up to 49.5, 46.8 and 37.1. Furthermore, $\Delta {{\mathrm{SUV}}_{\mathrm{max}}}_{(500-400)}\mathrm{\%}$, $\Delta {\mathrm{contrast}}_{(500-400)}\mathrm{\%}$ and $\Delta {\mathrm{CR}}_{(500-400) }\mathrm{\%}$ also receded by up to 16.0, 19.3, and 18.7 (Figs. 2, 4). Reynes-Llompart et al. [37] highlighted the relative difference of quantitative parameters in Q.Clear, when using a *β* value of > 500, plateaued.

Our results demonstrate a greater relative difference in CR and BV when using Q.Clear with a BGO scanner compared to the results of previous studies with an LYSO scanner. At an LBR 4:1, the $\Delta {\mathrm{CR}}_{(500-100)}$% in spheres with 10 mm and 22 mm diameters decreased by 128.8 and 15.8, respectively. Teoh's study filled the NEMA phantom with a 4:1 ratio and scanned it using an LYSO scanner. Their findings indicated that the $\Delta {\mathrm{CR}}_{(500-100)}$% in sphere with diameters of 10 mm and 22 mm was 100.2 and 8.4, respectively [14]. Our clinical investigation found that Q.Clear reconstruction yields greater improvement in quantitative parameters on BGO scanners compared to other scanners. Lesion ${\mathrm{SUV}}_{\mathrm{max}}$, SNR and SBR increased by 18.1%, 52.3%, and 16.9%, respectively, in ${\mathrm{Q}.\mathrm{Clear}}_{400}$ compared to OSEM. Lindström et al. conducted a clinical study using images obtained by an LSO scanner, showing that ${\mathrm{Q}.\mathrm{Clear}}_{400}$, in comparison with OSEM, resulted in 11%, 22%, and 12% increases in ${\mathrm{SUV}}_{\mathrm{max}}$, SNR and SBR, respectively[26].

Using a low penalizing parameter might increase noise, potentially leading to false positive enhancement when estimating lesion uptake or mistakenly identifying noise as lesions. Conversely, excessive smoothing in Q.Clear with higher *β* values could lead to reduced RC and, consequently, incorrect negative interpretations. Based on our phantom study, the optimal *β* value for small lesions ranges from 200 for LBR 2:1 to 300 for LBR 8:1, improving SUV while maintaining an acceptable noise level. For larger lesions, the optimal *β* value lies between 400 for LBR 2:1 and 500 for LBR 8:1 to enhance SNR. It is vital to recognize the variability in lesion ratios and the lack of preset lesion sizes within clinical studies. Detecting large lesions with high activity is relatively straightforward while pinpointing smaller lesions with low activity is more challenging. Therefore, if only one reconstruction is performed for diagnosis, a *β* value of 300 is recommended to achieve the most accurate interpretation of the images.

Numerous factors strongly influence the optimization of the Q.Clear algorithm in PET scanning. One pivotal factor is the application or purpose of the PET scan. Different clinical or research needs might necessitate varying levels of image resolution, noise suppression, or contrast enhancement. Contrast is another important element. Lesions with higher contrast (i.e., the pronounced difference in signal intensity compared to surrounding tissues) may require distinct optimization strategies than those with lower contrast. Images with different noise and varying lesion sizes demand unique optimization. The patient count in our study was below the desired number, and the limited range of lesion sizes constrained our analysis. Larger studies are needed to confirm our findings. Assessing the effects of Body Mass Index (BMI), scan duration, various PET applications, and injected dose on optimizing the *β*-factor for a broader patient population was not the focus of this investigation.

In conclusion, Q.Clear offers enhanced quantitative measurements while maintaining a noise level comparable to the OSEM algorithm. This results in superior image quality and lesion detection. The Q.Clear optimization depends on both lesion size and LBR. As lesion size and LBR decrease, the optimal *β* value follows suit. For rectal cancer cases, we suggest using ${\mathrm{Q}.\mathrm{Clear}}_{300}$ for smaller lesions and ${\mathrm{Q}.\mathrm{Clear}}_{500}$ for larger ones.

## Data Availability

The datasets used and/or analyzed during the current study are available from the corresponding author upon reasonable request.

## Abbreviations

TOF: Time of flight
BGO: Bismuth germanium oxide
LBR: Lesion-to-background ratio
NEMA: National Electrical Manufacturers Association
OSEM: Ordered subset expectation maximization
BSREM: Block sequential regularized expectation maximization
RC: Recovery coefficient
CR: Contrast recovery
SNR: Signal-to-noise ratio
SUV: Standard uptake value
SBR: Signal-to-background ratio
